# Applications of Biomaterials to Liquid Crystals

**DOI:** 10.3390/molecules18044703

**Published:** 2013-04-19

**Authors:** Kazuki Iwabata, Urara Sugai, Yasutaka Seki, Hirokazu Furue, Kengo Sakaguchi

**Affiliations:** 1 Department of Applied Biological Science, Tokyo University of Science, 2641 Yamazaki, Noda, Chiba 278-8510, Japan; E-Mails: sugai.urara.63@gmail.com (U.S.); y.seki1030@gmail.com (Y.S.); kengo@rs.noda.tus.ac.jp (K.S.); 2 Department of Industrial Chemistry, Tokyo University of Science, 2641 Yamazaki, Noda, Chiba 278-8510, Japan; E-Mail: hfurue@rs.noda.tus.ac.jp

**Keywords:** liquid crystal, biomaterial, DNA, peptide

## Abstract

Nowadays, chemically synthesized proteins and peptides are attractive building blocks and have potential in many important applications as biomaterials. In this review, applications of biomaterials to thermotropic liquid crystals are discussed. The review covers the improvement of the performance of liquid crystal displays using liquid crystal physical gels consisting of a liquid crystal and amino acid-based gelators, and also new functionalization of liquid crystals. Moreover, the influence of DNA, which is one of the more attractive biomaterials, dispersed in thermotropic liquid crystals and its potential use in the liquid crystal industry is described. In addition, we found interesting results during electrooptical measurements of liquid crystals doped with DNA, and explain them from the point of view of biological applications. These recent approaches suggest that these biomaterials may be applicable in the electronic device industry and should be considered as an interesting material with their physical properties having the potential to create or refine an industrial product.

## 1. Introduction

In recent years chemically synthetized peptides and proteins have attracted the attention of researchers working in various areas of science. One of the more interesting of these new research areas is the exploitation of peptides for the assembly of nanomaterials [1]. Peptides are particularly attractive as molecular building blocks in the bottom-up fabrication of supramolecular structures based on self-assembly and have potential in many important applications. Cavalli *et al*. demonstrated the importance of amphiphilic peptides as molecular building blocks for nanostructures through discussion of several examples of applied nanomaterials [2]. There are also many reports which describe applications of peptide-based self-assembled structures [3,4,5]. Along with peptides, much attention has also been focused on the rational design of self-assembled nanostructures using biomaterials such as DNA, RNA, and polypeptides [6,7,8,9,10,11,12,13,14]. Furthermore, as examples of the research into physical and chemical properties of the materials, Lowik *et al*. give an introduction into the field of stimulus-responsive peptide-based materials [15]. They have tried to categorize them according to the stimulus the materials are responsive to, these being pH, temperature, metal ions, enzymes and light.

The techniques and the knowledge produced by these developments have spread to other areas. One example is that there have been moves to apply biomaterials to electronic devices. Amyloid fibrils are one of several self-assembling peptide systems that are attracting increasing interest for molecular electronics applications [12,16,17]. Del Mercato *et al*. demonstrated that nanofibrils can sustain significant electrical conduction in the solid state at ambient conditions and have remarkable stability [17]. Recently, with the development of the technology of self-assembled nanostructures using biopolymers, biomaterials seem to be ideally suited to improve the performance of photovoltaic and organic field effect transistor devices [18]. By the attachment of an oligothiophene derivative to the side chain of L-lysine and subsequent polymerization to the corresponding poly-L-lysine derivative, the resulting compound provided an improved photovoltaic and organic field effect transistor device [18].

In this review, we give other examples of biomaterials applied to electronic devices, especially liquid crystals (LCs), and show their possibilities. Since the pocket calculator with LC display was first introduced in 1973, liquid crystals are now used in flat-panel televisions across the globe [19]. Even now, there are a lot of studies to open up the potential of liquid crystals. We describe some of these approaches in this review.

## 2. What are Liquid Crystals?

**Figure 1:**
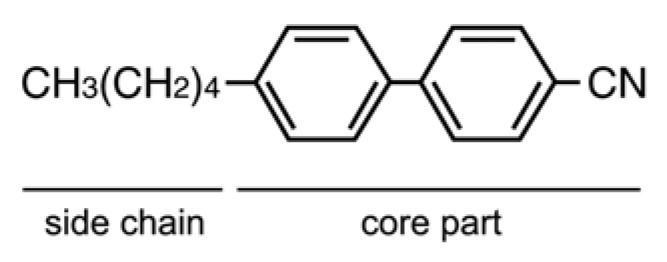
An illustration of the structure of 5CB.

Liquid crystals may flow like a liquid, but the molecules in the liquid are arranged and/or oriented in a crystal-like manner. There are two generic classes of liquid crystals: those whose transitions are driven by thermal processes, known as thermotropic liquid crystals, and those strongly influenced by solvents, known as lyotropics. Many thermotropic LCs exhibit a variety of phases as the temperature is changed. For instance, a particular type of LC molecule may exhibit various smectic and nematic phases as the temperature is increased. Thermotropic liquid crystal materials have characteristics related to their molecular structure, which consist of two parts, namely the core and side chain. The core part is a rigid body which brings shape anisotropy to the molecule, and the side chain part is a flexible region which gives mobility. Figure 1 shows a typical thermotropic liquid crystal, 4-cyano-4'-pentylbiphenyl (5CB). These liquid crystal materials are being constantly developed and improved, and used industrially in various ways such as in displays, film, drugs and medicines. Kato *et al*. and Gao *et al*. have recently reported that dendritic oligopeptides can act as useful building blocks for chiral supramolecular liquid crystals [20,21,22].

**Figure 2:**
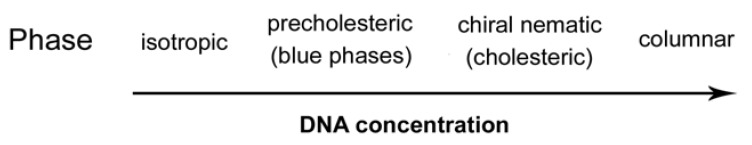
The schematics of nucleic-acid based liquid crystal phases.Adapted with permission from [27].

In contrast, lyotropic liquid-crystal materials may not necessarily possess shape anisotropy, but rather self-assemble depending on their concentration in a solvent. There is a connection between biomaterials and liquid crystals [23]. According to Robinson, pure DNA forms liquid-crystalline phases *in vitro* [24]. Also, it has been known that DNA exhibits: (i) precholesteric or blue phases, (ii) chiral nematic (also known as cholesteric), and (iii) columnar phases, on increasing concentration (Figure 2). This has been determined from *ex vivo* studies using aqueous solutions [23,24,25,26,27,28]. Furthermore, DNA forms a hexagonal columnar phase in association with cationic lipids, which is expected to be an alternative carrier for gene therapy without using a virus [29,30,31]. Powell *et al*. described how the formulation of Deterelix, a hydrophobic Luteinizing Hormone Releasing peptide, rapidly formed nematic peptide liquid crystals of undulose extinction when in aqueous solution [32]. While lyotropic LCs are very widely used in cosmetics and beauty care and have been explored for their potential applications, in this review, we focus on thermotropic liquid crystals because there are few reports describing the application of biomaterials to thermotropic LCs although thermotropic liquid crystals applied to LCDs have been well-researched and are already in commercial use [33].

## 3. New Functional LC Modulated with Peptide

Recently, there have been new approaches to producing new functional LC materials which induce amino acids or peptides. In the traditional development process of liquid crystal molecules used for displays, there is no need to consider the introduction of functional groups which induce strong intermolecular interactions, such as hydrogen bonds, electrostatic interactions, and charge transfer interactions, but utilization of these functional groups cannot be ignored when it comes to the evolution and development of novel applications of liquid crystals. 

**Figure 3:**
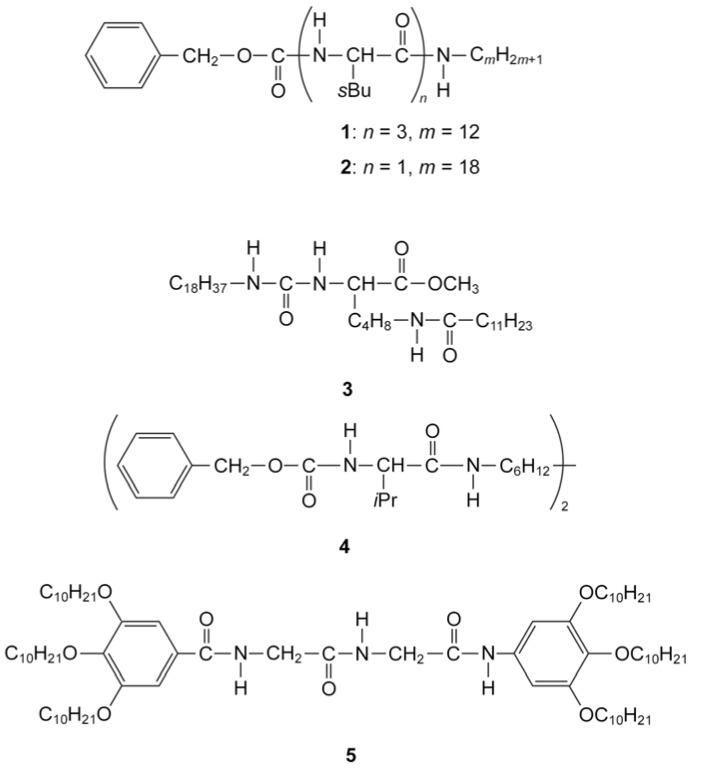
Chemical structures of gelators.

Kato and collaborators have succeeded in the preparation of LC physical gels with new structures and functions [34,35,36,37,38,39,40,41,42]. LC physical gels are composites consisting of a liquid crystal and a small amount of self-assembled fibers [34]. The fibrous materials are simply formed in the liquid crystals by self-assembly of low-molecular-weight compounds (gelators) which have amino acid (or a derivative) moieties in their chemical structure, a process which is driven by hydrogen-bonding. The number of amino acid moieties in the gelators has a large effect on the gelation ability and the electro-optical behaviour of the resulting LC gels (compounds **1** and **2** in Figure 3) [35]. These materials are soft solids retaining liquid-crystalline properties (compounds **1**–**4** in Figure 3). 

Such gels show thermoreversible sol-gel transitions which arise from the association and dissociation of intermolecular interactions between the gelators [34]. For LC physical gels, the LC and gelators show individual transitions. By using the gelators, they have found that physical gelation of liquid crystals improved the response speeds of liquid crystals to electric fields [36,37]. They explained that the existence of the finely dispersed aggregates of the gelator induces LC alignment states that are both metastable and responsive. Moreover, the interactions between the liquid crystals and the fibrous aggregates properly weaken an alignment force to the LC molecules conveyed from the rubbed-polyimide surfaces, which leads to the faster response [36]. Another gelator which is a monodisperse homomeric dipeptide also improves the dynamic characteristics of the electro-optical switching of the device employing these gels (compound **5** in Figure 3) [43,44]. These effects suggest not just the improvement of response speed, but also two possibilities for new applications of liquid crystals: light scattering displays and memory. Firstly, because of the formation of random dispersion of gelators, nematic LC gels was found to be suitable for light scattering displays switching electro-optically between turbid and transparent states [35,38,39,40,41]. When the fibrous assembly of a gelator occurs at a temperature higher than the isotropic-anisotropic transition temperature of a liquid crystal component, a randomly dispersed fiber network is formed in the isotropic medium [34]. As no polarizer is needed in light scattering electrooptical displays, the brightness of the displays becomes higher. Due to its simple device structure, light scattering electrooptical switching is expected to be useful for large area display [35]. Secondly, they also found that rewritable bistable nematic materials exhibiting light-scattering and transparent states have been achieved by a combination of thermal and electric stimuli [42]. That is to say, it can be used as a memory. Association of a gelator in a liquid-crystalline phase induces the formation of an oriented self-assembled fiber network, resulting in an oriented liquid-crystalline gel after removal of the electric fields. In this case, the liquid crystal serves as a template [34].

These new initiatives are based on the particular characteristics of amino acids. Amino acids are very functional molecular frames when designing a gelator because they have the advantages of easy chemical modification and polymerization, bringing chirality to the compound, and providing hydrogen-bond interactions. Utilization of non-natural amino acids or peptides might provide clues to help in the development of alternative functional liquid crystals and allow them to be put to practical use.

## 4. Liquid Crystals and Doping Agents

**Figure 4:**
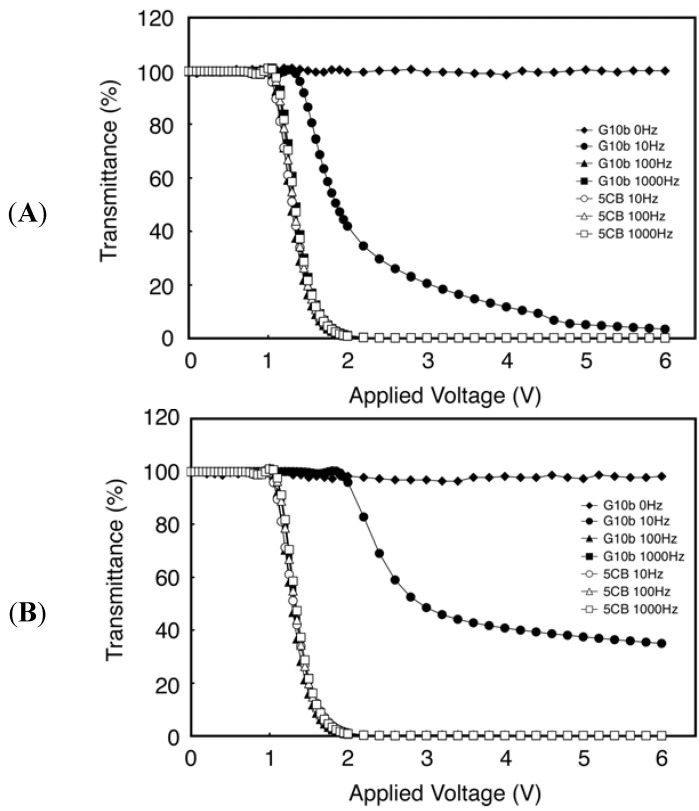
Electrooptic characteristics of LC cells. The frequency of the operating voltage is used as a parameter. Open dots indicate the experimental data from LC cell doped with (**A**) G10b (100 µM) or (**B**) G10b (500 µM), and filled dots indicate that from LC cell with pure 5CB, respectively. Adapted with permission from [45]. Copyright 2011 The Japan Society of Applied Physics

As described above, new applications have been created by introducing amino acids or derivatives into the chemical structures of liquid crystals. Kato and collaborators have succeeded in creating new liquid crystal functionality by making gelators which contain amino acids or derivatives. In contrast we are attempting to find ways to add new function to liquid crystals by dispersing the biomaterials themselves. As a first step, we have focused on DNA because of its stability and structural simplicity. We have prepared liquid crystal samples doped with oligonucleotide DNA (ssDNA or dsDNA) and investigated the effects. We have found two favorable characteristics. Firstly, by measuring the voltage-transmittance (V-t) response, we have succeeded in determining the characteristics of the frequency modulation (FM) response in twist nematic liquid crystal displays (TN-LCDs) doped with guanine 10-base (G10b; 5'-GGGGGGGGGG-3') at concentrations of 100 and 500 μM, where 5CB (nematic LC) was used as the host medium. Figure 4 shows the FM response result. Thus, it is possible to drive the LCD device by changing the frequency of the applied voltage without changing the amplitude [45]. Shiraishi and coworkers also reported that nematic LCDs doped with metal nanoparticles, such as Pd, Ag, and Ag-Pd composites, exhibit an FM electro-optical response [46,47,48,49,50]. Applying an electrical field with changing frequency these LCDs doped with metal nanoparticles show a faster switching response than undoped-conventional TN-LCDs [48]. The rapid change in the dielectric anisotropy caused by changing frequency would seem to explain the fast response [48]. We expect that this characteristic would find application as a new control method to improve the response speed of LCDs. Secondly, measurement of the Circular Dichroism (CD) spectra demonstrates that dsDNA produced a twist deformation of the liquid crystal system as determined by a microscopic analysis of the CD data, while ssDNA does not [51]. Furthermore, it should be noted that the magnitude of twisting power is different for the A-T and G-C DNA pairs [51]. This twist deformation might originate from the molecular conformation of dsDNA. Due to the cooperative nature of liquid crystal ordering, however, a small amount of chiral dopant in an otherwise achiral mesophase is often enough to select out one domain handedness, making the system chiral overall. The results of the CD spectra suggest that dsDNA may be of use as a chiral dopant that regulates the twist of liquid crystals. These observations are a result of the use of DNA and as such it may be difficult to create variations in such materials because DNA consists of only four kinds of base units, namely, adenine, thymine, cytosine, and guanine. In contrast, peptides are attractive compounds because they can be designed with respect to the various amino acids. Typical commercially available chiral compounds have rather low helical twisting powers, mostly due to shape, size, polarisation, or conformational incompatibility with the LC host [52]. Therefore, a favorable peptide could possess low-molecular-weight, dipole moment, inter- or intra-molecule conformation, or a polymer structure.

## 5. Biological Applications

**Figure 5:**
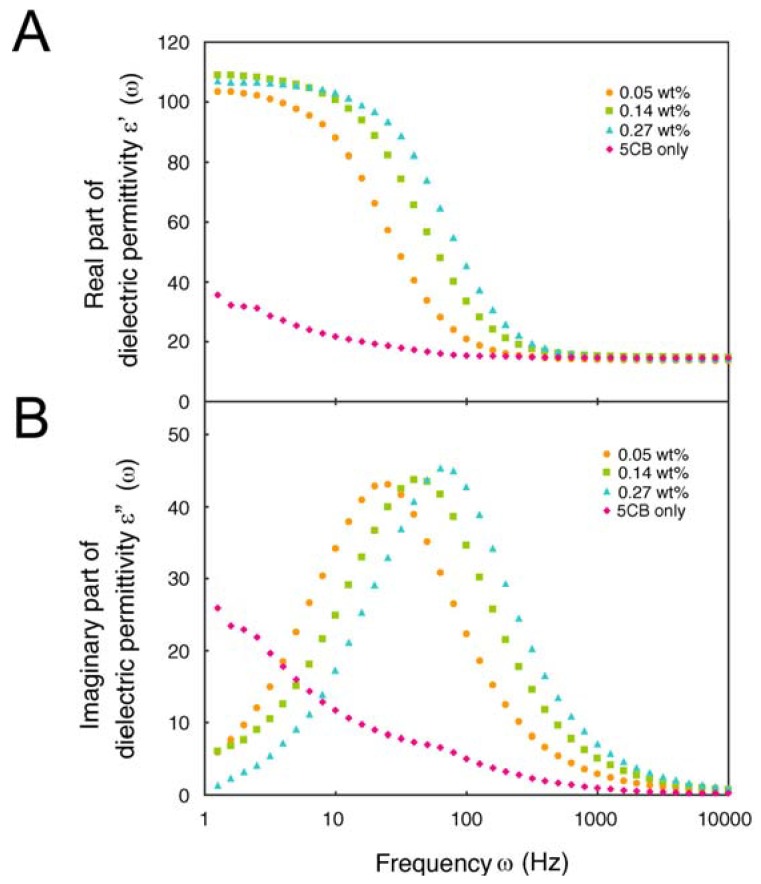
Dielectric relaxation dependent on the concentration of C10b. The dielectric permittivity of 5CB doped with various concentrations of C10b was analyzed; orange circles, green squares and blue triangles indicate the experimental data from 5CB doped with 200 µM (0.05 wt%), 500 µM (0.14 wt%) and 1000 µM (0.27 wt%) C10b, respectively. The red rhombic dots indicate the data from pure 5CB as a reference. (**A**) shows the real part of the dielectric permittivity ε’ and (**B**) shows the imaginary part of the dielectric permittivity ε”. Adapted with permission from [53]. Copyright 2010 The Japan Society of Applied Physics.

During a series of measurements, some interesting results have emerged and these will be discussed in relation to their biological applications. First, we measured the dielectric properties of NLCs doped with ssDNA (A10b, C10b, G10b, and T10b) [53]. The result shows that 5CB doped with oligonucleotide exhibits dielectric relaxation from 10^1^ to 10^2^ Hz, as shown in Figure 5.

This suggests that the oligonucleotide exists as a polar molecule in the liquid crystal media. Kobayashi *et al*. reported the same phenomenon using nematic liquid crystals doped with metal nanoparticles, and explained it with their crystal model and by an equivalent circuit approach [54]. We also applied this theory to our experimental results. Consequently, we compared the electric conductivities of the four bases and found that the values for adenine and thymine were distinct from those of cytosine and guanine. This result suggests that the difference in electrical conductivity contributes to the number of hydrogen bonds. This contribution also affects the dielectric permittivity. A number of experimental and theoretical studies have been performed to clarify the electrical conducting properties of DNA duplexes [55,56,57,58,59,60,61,62,63,64,65,66,67,68,69,70,71,72,73,74,75,76,77,78,79,80,81,82,83,84,85,86].

**Figure 6:**
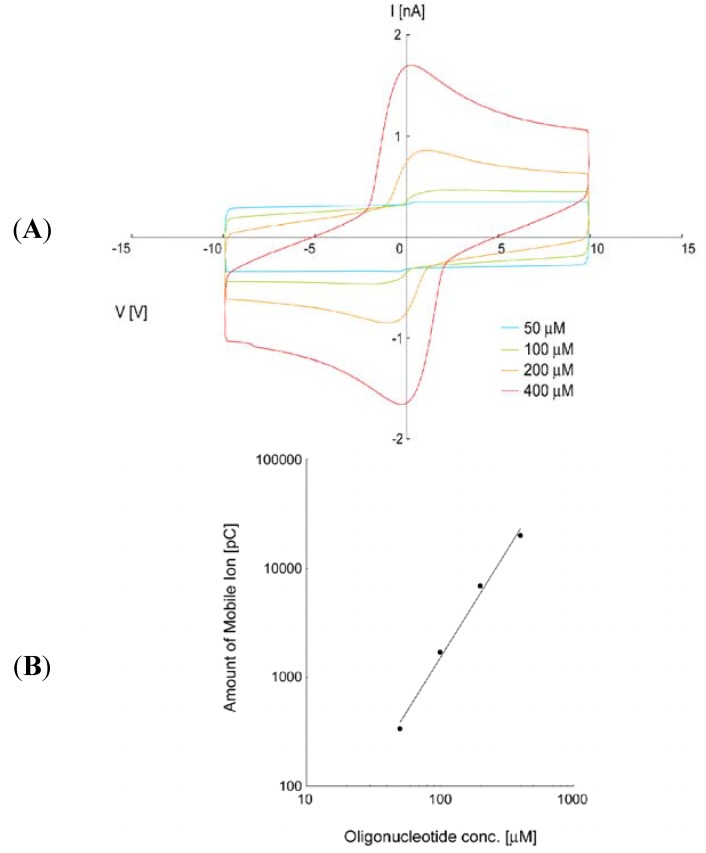
Concentration characteristics of LC cells doped with G10b. (**A**) Each line represents the I-V curve of the LC cell doped with G10b at different concentrations; red line: 400 µM, orange line: 200 µM: green line: 100 µM, and blue line: 50 µM. (**B**) Shows the relationship between concentration of G10b and ion density of LC cells, calculated from the peak at positive voltage application. The horizontal and vertical axes of our measurement results are concentration of G10b in LC cells and ion density of LC cells, respectively.

Second, by measuring ion density, we examined whether ion density was affected by the identity of the DNA base. Inoue *et al.* described an analysis method which can judge the polarity of mobile materials in an LC layer from the asymmetric characteristics of a voltage *vs.* current (I-V) curve, known as ion density measurement [87]. From this curve, we could estimate the ion density of a LC cell. We prepared LC (MLC6884, Merck) samples doped with various oligonucleotides (A10b, G10b, C10b and T10b) at different concentrations and fabricated LC cells. As a result, we obtained a linear correlation between various concentrations of oligonucleotides and ion density of LC cells on a graph using a logarithmic scale. Figure 6 shows the correlation characteristics of LC cells doped with G10b. 

It should be noted that there is a difference between the fitting parameters [88]. This result suggests that ion density is affected by the identity of DNA base. Taking the results together, we found that oligonucleotides show a sequence specific correlation between concentration and the amount of mobile ions in LC cells [88]. These studies suggest that dielectric and ion density measurements could open new opportunities for analysis of the electrical properties of biological molecules. It is an important point to note that that the measurement using liquid crystal can completely exclude the effect of water molecules, which always interferes with electrical analysis.

Incidentally, there have been some reports describing early successes for new liquid-crystal material applications with notable biomedical and biological implications. Devices and configurations based on liquid-crystal materials are being developed for spectroscopy, imaging and microscopy, leading to new techniques for optically probing biological systems [89]. Also, a series of investigations have reported biosensors which use the interface of thermotropic liquid crystals as a detection system [90]. One example is that Brake and colleagues reported that the spontaneous assembly of phospholipids at planar interfaces between thermotropic liquid crystals and aqueous phases gives rise to an ordering transition of the liquid crystals [91]. The system based on the ordering transition of LC interfaces is available as optical sensors to monitor for binding events. A series of recent reports has demonstrated that a rich diversity of amphiphilic molecules, such as phospholipids, surfactants, and polymers can assemble at interfaces formed between thermotropic liquid crystals and immiscible aqueous phases [92]. With advancing technology, the preparation of peptide-modified interfaces has received a great deal of attention due to the potential utility of these interfaces for monitoring enzymatic activities, controlling cellular behaviors, and manipulating peptide-protein interactions [93,94]. Lundgren and colleagues developed a liquid crystal pixel array for a high number of signal discrimination [95]. The results described here will be the first step in utilizing liquid crystals as new biotechnological tools. In the near future, moreover, we expect the techniques to be applicable for investigating plasma membranes, liposomes and other domains or structures which show liquid crystallinity within cells.

## 6. Conclusions

In this review, we describe some possibilities of biomaterials applied to the liquid crystal industry. Physical gelation of liquid crystals by an amino acids-based gelator improves the performance of LCDs. DNA could be a chiral dopant and provide the new method of LCD regulation. New functionalities such as scattering displays and memory are added to liquid crystal by gelators. On the other hand, measurements of liquid crystal doped with biomaterials are potentially applicable as new biotechnological tools. These new initiatives make us feel the potential of biomaterials not only for medical and biological application but also for electronic device industrial usage. The research achieving the improvement of photovoltaic and organic field effect transistor device shows a good example of biomaterials’ availability. Although we only refer to liquid crystal examples, these biomaterials may have applicability in industrial use if they are considered as an interesting material with the potential to create or refine an industrial product by developing processes that take advantage of their electrical, physical, and structural properties.
